# A Hybrid Transformers-based Convolutional Neural Network Model for Keratoconus Detection in Scheimpflug-based Dynamic Corneal Deformation Videos

**DOI:** 10.18502/jovr.v20.17716

**Published:** 2025-06-18

**Authors:** Hazem Abdelmotaal, Rossen Mihaylov Hazarbasanov, Ramin Salouti, M. Hossein Nowroozzadeh, Suphi Taneri, Ali H. Al-Timemy, Alexandru Lavric, Hidenori Takahashi, Siamak Yousefi

**Affiliations:** ^1^Department of Ophthalmology, Assiut University, Assiut, Egypt; ^2^Hospital de Olhos-CRO, Guarulhos, SP, Brazil; ^3^Department of Ophthalmology and Visual Science, Paulista School of Medicine, Federal University of S ao Paulo, SP, Brazil; ^4^Poostchi Ophthalmology Research Center, Shiraz University of Medical Sciences, Shiraz, Iran; ^5^Ruhr University, Bochum, Germany; ^6^Zentrum für Refraktive Chirurgie, Muenster, Germany; ^7^Biomedical Engineering Department, Al-Khwarizmi College of Engineering, University of Baghdad, Iraq; ^8^Computers, Electronics and Automation Department, Stefan cel Mare University of Suceava, Romania; ^9^Department of Ophthalmology, Jichi Medical University, Tochigi, Japan; ^10^Department of Ophthalmology, University of Tennessee Health Science Center, Memphis, TN, USA; ^11^Department of Genetics, Genomics, and Informatics, University of Tennessee Health Science Center, Memphis, TN, USA

**Keywords:** Artificial Intelligence, Corneal Imaging, Deep Learning, Keratoconus, Scheimpflug Imaging

## Abstract

**Purpose:**

To assess the performance of a hybrid Transformer-based convolutional neural network (CNN) model for automated detection of keratoconus in stand-alone Scheimpflug-based dynamic corneal deformation videos (DCDVs).

**Methods:**

We used transfer learning for feature extraction from DCDVs. These feature maps were augmented by self-attention to model long-range dependencies before classification to identify keratoconus directly. Model performance was evaluated by objective accuracy metrics based on DCDVs from two independent cohorts with 275 and 546 subjects.

**Results:**

The model's sensitivity and specificity in detecting keratoconus were 93% and 84%, respectively. The AUC of the keratoconus probability score based on the external validation database was 0.97.

**Conclusion:**

The hybrid Transformer-based model was highly sensitive and specific in discriminating normal from keratoconic eyes using DCDV(s) at levels that may prove useful in clinical practice.

##  INTRODUCTION

Keratoconus (KC), the most common ectatic corneal disease, is characterized by progressive corneal steepening and thinning due to deficits of structural integrity. These deficits lead to irregular astigmatism and myopia with deterioration of visual acuity and vision-specific quality of life.^[1,2]^ Early diagnosis of KC is essential to halt disease progression and avoid keratectasia after a refractive procedure.^[3]^ Corneal topography or tomography systems have become crucial tools in clinical practice for early detection of alterations in corneal shape, and for assisting the examiner in KC classification and monitoring.^[4]^ However, these instruments do not measure the mechanical stability of the cornea, which is thought to be reduced in KC, thereby triggering stromal thinning and steepening.^[5]^ As a result, there has been increasing interest in measuring biomechanical properties of the cornea in vivo before the onset of topographic or tomographic changes.^[6]^ The Corneal Visualization Scheimpflug Technology (Corvis ST, Oculus Optikgeräte GmbH, Wetzlar, Germany) is a relatively new instrument that records the ocular deformation response induced by a constantly defined air puff using an ultra-high-speed Scheimpflug camera. The captured images are analyzed by the Corvis ST software to produce an estimate of intraocular pressure (IOP) as well as several dynamic corneal response (DCR) parameters that provide a detailed assessment of corneal biomechanical properties. These parameters can be used to separate normal (N) from ectatic corneas.^[7,8,9]^ A video function uses these images to release a slow-motion video of the corneal deformation reaction in response to the air pulse (4330 frames/second). This highly precise video permits a unique, viewable biomechanical analysis of the cornea.^[10]^

Convolutional neural networks (CNNs), the de facto deep learning operator for computer vision, process visual information represented as an array of pixels. Although CNN-based models have shown outstanding performance in several domains, the design of the convolutional layer imposes locality, via a limited receptive field, and translational equivariance, via weight sharing. Both these properties prove to be crucial inductive biases when designing models that operate over images. However, convolutional kernels are inherently constrained to capture local contexts, which prevents capturing long-range interactions between the semantic concepts of objects within images.^[11]^ On the other hand, self-attention has emerged as a popular architecture for sequence-to-sequence modeling, but it has primarily been applied in natural language processing (NLP) and generative modeling. The main concept of self-attention is to produce a weighted average of values that, unlike the pooling or the convolutional operators, are produced dynamically via a similarity function between hidden units. As a result, the interactions between the input signals depend on the signals themselves rather than their relative location, as in convolutions. This allows self-attention to capture long-range dependencies without the requirement for increasing the number of parameters.^[12]^

In videos, long-range interactions occur between distant pixels in spatiotemporal space. Thus, attention-based architectures are an intuitive choice for modeling these long-range contextual relationships in video.^[11]^

The success of attention-based NLP models has recently inspired approaches in computer vision to integrate Transformers into CNNs to improve their performance,^[13]^ and some attempts to replace convolutions completely.^[14]^ Recently, the video vision Transformer (ViViT), a pure Transformer-based architecture, achieved state-of-the-art performance on multiple standard video classification benchmarks. However, as Transformers lack some of the inductive biases of convolutional networks, they applied several strong regularization strategies to overcome overfitting on smaller datasets.^[15]^

To overcome the challenges inherent to the convolution and Transformer architectures, we developed a hybrid Transformer-based model for Corvis-ST video classification. The adopted model comprises a CNN augmented by incorporating self-attention. This approach leverages the respective strengths of each operation, using convolutions to extract video frame features and transformers to relate high-level concepts. Finally, these augmented feature maps are used for KC detection in standalone Corvis ST videos without using corneal topography or tomography data.

##  METHODS

The Institutional Review Boards of the Hospital de Olhos-CRO, Guarulhos (as the affiliate center), the Federal University of So Paulo-UNIFESP/EPM (as the coordinator center), and the Salouti Eye Center in Iran gave their approval for this study, which was carried out in accordance with the Declaration of Helsinki and its later amendments. Both IRBs were exempt from receiving form of consent as only de-identified archival data was prospectively analyzed. To utilize the data, contributing parties signed corresponding data usage agreements. We retrospectively analyzed datasets with records of 821 nonconsecutive, refractive surgery candidates, and patients with unilateral or bilateral KC, in these two centers located on two different continents, which ensured the ethnic diversity of subjects during testing our approach to distinguish between normal (N) and keratoconic (KC) corneas. Data were de-identified before further processing in Brazil and Iran after subjects provided informed written consent to participate in the study. A total of 275 subjects (127 N and 148 KC) were enrolled from the Hospital de Olhos-CRO, Guarulhos (Brazil-Dataset 1; collected from January 2020 to December 2021), and 546 subjects (285 N and 261 KC) were enrolled from the Salouti Eye Center (Iran-Dataset 2; collected from January 2019 to December 2021). Authors had no access to the patient information during data collection.

All participants received a comprehensive ophthalmic examination, including Scheimpflug-based corneal tomography using the Pentacam HR and corneal biomechanical assessment using the Corvis ST (Oculus Opikgeräte GmbH).

The inclusion criteria for N cases were: (1) having normal cornea on the general eye examination in both eyes, including normal slit-lamp biomicroscopy; (2) corrected distance vision of 20/20 or better; (3) subjective normal topography; and (4) tomography exams without prior topical medications except artificial tears in both eyes.

The criteria for clinical diagnosis of ectasia included clear signs of KC in both eyes derived from Scheimpflug tomography (adapted from the keratoconus Amsler– Krumeich^[16]^ stages grading by the Pentacam software), as approved by an experienced cornea specialist in each center (RH and RS) with at least stage 1 topographical KC classification (TKC) with or without slit-lamp findings (e.g., cone-shaped deformity, Munson's sign, Rizutti's sign, Vogt's striae, Fleischer ring, or apical thinning).^[1]^

All study participants were requested to discontinue wearing contact lenses for 10 days. Patients with a history of ocular surgery, collagen vascular disease, or diabetes mellitus were excluded from the study. Moreover, to confirm the diagnosis of KC or N, all cases from each clinic were blindly reviewed by a third-party anterior segment expert (ST) to confirm inclusion criteria.

Measurements in each center were performed by the same experienced examiners using the same software and hardware, captured by automatic release to ensure user independence, and only examinations with acceptable quality scores for proper analysis were included. For each participant, only one eye was randomly included in the analysis to avoid bias due to the relationship between bilateral eyes in the subsequent analysis.

The data that support this study's findings are not openly available due to the inclusion of patient information and reasons of sensitivity. However, data can become available from the corresponding authors upon reasonable request and signing the corresponding data use agreement.

### Dynamic Corneal Response (DCR) Parameters

The Corvis ST measures the corneal response to an applied predefined air impulse (held constant for every measurement for every device) using an ultra-high-speed Scheimpflug camera that captures 4330 frames per second for only 33 ms and eventually produces a video with 139 frames. By analyzing the whole process of the corneal dynamic response recorded, DCR and related corneal thickness (pachymetry) parameters are calculated. Corvis-ST data offers a comprehensive set of parameters, including deflection and deformation factors. We carefully selected a subset of the top nine best-performing DCR parameters and summary indices concerning KC detection for inclusion in our analysis. ${}^{\left[6,7,8,9,10\right]}$ The following parameters were included in our analysis: A1 velocity, which is the speed of corneal apex at first applanation (A1); A1 deflection amplitude in mm (A1 DA [mm]), which is determined as the displacement of the corneal apex relative to the initial state at first applanation without the maximum whole eye movement quantification; Deflection amplitude ratio at 2 mm (DA Ratio Max [2 mm]), which is the ratio between the deformation/deflection amplitude at the apex and the average deformation/deflection amplitude measured at 2 mm from the center;^[7]^ PachySlope (µm), calculated as peripheric (8 mm horizontal) pachymetry/apex pachymetry; Ambrósio relational thickness to the horizontal (8 mm) profile (ARTh), which is based on the thickness profile in the temporal nasal direction (lower values indicate a thinner cornea and/or faster thickness increase toward the periphery^[8]^); Integrated Radius [mm ∧ -1], which is the integrated area under the radius of the inverted curvature during the corneal highest concavity phase.^[7]^

The stiffness parameter at first applanation (SP-A1) is defined as the resultant pressure divided by the deflection amplitude at the first applanation. The resultant pressure is defined as the adjusted air pressure at A1 minus the biomechanically corrected intraocular pressure (bIOP) value. The adjusted air pressure represents the load of the air pressure (calculated by converting the spatial and temporal velocity profiles of the air puff to pressure) impinging on the cornea at A1.^[7]^ The bIOP was derived by finite element simulation, considering the influence of central corneal thickness (CCT), age, and DCR parameters. This value was validated both experimentally and clinically.^[17]^ CBI includes several DCR parameters in addition to ARTh. This combined index is calculated by logistic regression analysis to determine the cut-off value that discriminates N from KC classes.^[8]^ The TBI is generated by combining DCR and tomographic parameters using the leave-one-out cross-validation technique implemented by a random forest classifier to determine the cut-off value for detecting KC and N eyes.^[9]^

Data from the Pentacam HR and Corvis ST were exported to a custom spreadsheet and linked to the related videos and participants' demographic data. Each video was tagged by a serial number and the specific class (N or KC).

### Corvis ST Video Frames Preprocessing

A total of 821 high-quality slow-motion video clips from 821 eyes, showing the corneal deformation resulting from a constant air pulse, were exported along with related demographic, tomographic, and biomechanical data in both study centers. Each video is a synchronized release of the cross-sectional corneal image covering the horizontal 8.8 mm of the cornea, showing the sequence of corneal shape transformation from convex to concave, passing through the first applanation state. When the air puff peaks, the cornea is at its highest concavity. When the air puff ceases, the cornea returns to its original natural shape, passing through the second applanation state, and the video frame capturing ends. During the recording process, a blue LED light (470 nm wavelength, ultraviolet-free) illuminates the imaged area, and the dispersed light from the cornea is recorded.^[7]^ Recorded videos were sampled in audio video interleaved (AVI) format at a resolution of 576 × 214 at 13 frames per second, yielding a video length of 10 seconds. All videos were resized to a resolution of 224 × 224 using the OpenCV library (version 4.5.4, http://opencv.org). As all videos have the same length, no padding was required. Dataset 2 videos were used for training and internal validation (70%) and testing (30%) of the hybrid model. Dataset 1 was used for external validation of the model performance (external validation dataset).

### Hybrid Model Architecture

**Figure 1:**
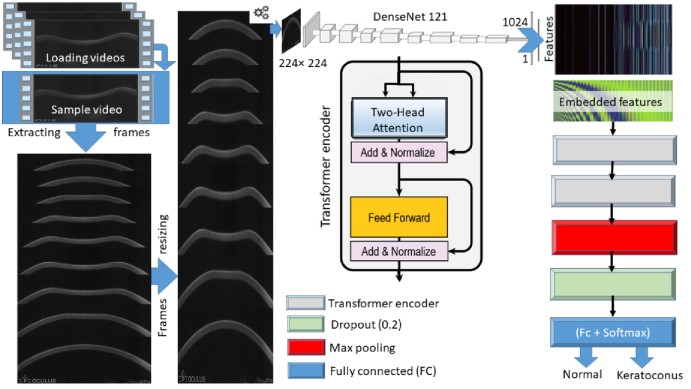
Overview of the hybrid model. After loading each Corvis ST video, we used the Densenet121 as a feature extractor to get a 2D feature map representing features extracted from all frames, then linearly embedded each map, added position embedding, and fed the resulting sequence of vectors to a stack of two standard two-head Transformer encoders as a flattened 1D sequence of token embedding of the same size as the Transformers latent vector size (dimension = 1024). This is followed by Maxpooling 1D, a Dropout layer (rate = 0.2), and a fully connected (two nodes) classification head with SoftMax activation function.

An overview of the hybrid model design is depicted in Figure 1.

### The first component of the hybrid model (feature extractor CNN)

For extracting feature maps from each Corvis ST video, we adopted a pre-trained DenseNet121, which has been shown to have better feature use efficiency and outperform ResNet with fewer parameters.^[18,19]^ The model was initialized with the loaded ImageNet weights.^[20]^ The output (classifying) layer was truncated, and the extracted 1024 features per frame were squeezed to get a 139 × 1024 feature map per video.

### Second (classification) component of the hybrid model

After implementing, tuning, and evaluating several design variants, the following classification architecture was proposed:

#### Positional encoding

As self-attention layers that form the basic blocks of a Transformer are order-agnostic and the feature maps are extracted from ordered sequences of frames, an additional vector is added to the pre-computed feature maps to account for the frame order of feature maps elements by simply embedding the positions of the frames with an embedding layer. This positional embedding follows a specific pattern that the model learns in order to determine the position of feature vectors relative to the source frame.

#### Transformer encoder

The embedded feature maps constitute the input sequence projected to the Transformer dimension, formed of two stacked Transformer encoder layers. A Transformer encoder layer receives an embedded sequence of features that are first encoded in parallel to generate key, query, and value representations. The keys and corresponding queries are combined by a compatibility function to generate attention weightings assigned to each value that capture how each feature relates to the others in the map. These attention weightings are then used to scale the values, retain focus on the important features, and drown out the irrelevant ones. This can effectively augment feature maps by adding information about the relative importance of other features relative to every single feature in the maps, thus overcoming the spatial constraints imposed by convolutions. In our approach, we performed a two-head attention function.^[12]^

#### Hidden and output layers

The augmented feature maps output from the second Transformer encoder layer undergo dimensionality reduction in the Global Max pooling layer that down-samples the input representation by taking the maximum value over the frame number (1–139) dimension. This is followed by one Dropout layer with a 0.2 dropout rate, and the final fully connected (output/classifying) layer containing two fully connected neurons with SoftMax activation to provide the likelihood of N or KC classes.

### Model training

The model was trained using sparse categorical cross-entropy as the loss function.^[21]^ The optimization was performed using a stochastic gradient descent (SGD) optimizer with Nesterov momentum, an initial learning rate of 0.1, a momentum of 0.9, and a weight decay of 4e-5. The model was trained for 200 epochs, with a scheduled exponential learning decay rate of 0.9.^[22]^

### Model testing 

After training, the model's performance was assessed on the test subset feature maps. We used several objective metrics to evaluate the model, including accuracy, recall, precision, specificity, F1 score, confusion matrix, receiver operating characteristic curve (ROC), and area under the curve (AUC).^[23]^

### Model's variants and ablation

We conducted limited experiments to evaluate the importance of model components by exploring the influence of ablation/variation of model architecture (feature extraction backbone model, number of Transformer encoders, dropout, and fully connected layers, and number of attention heads) and frame sampling (number of input frames used).

### Final model 

To get the finalized model, the whole Dataset 2 feature maps (all available training and testing subsets) were used to repeat model training, applying the same parameters and with the aim of boosting model performance on external validation (Dataset 1).

### Model performance benchmarking

To obtain benchmark performance metrics that allow comparison of the model performance in KC detection, we used the selected DCR parameters and indices from Dataset 2 for training nine Naive Bayes Classifiers (NBCs) to solve this binary classification problem.^[24]^ The corresponding parameters in Dataset 1 were used to validate the classification performance of these trained NBCs. Similarly, the performance of the finalized trained model on feature maps in Dataset 1 was compared to the NBCs' performance using the ROC curve, AUC, and detection error tradeoff (DET) curve.^[23]^ A pairwise comparison of the AUC of our algorithm with the AUC(s) of these DCR parameters and indices was performed using the method described by DeLong et al.^[25]^

### Correlations

The correlation between the studied DCR parameters and indices with age, CCT, and IOP was evaluated using the Spearman rank correlation coefficient.

### Inspecting processed data

To better understand the role of Transformers in the hybrid model, we employed principal component analysis (PCA) for dimensionality reduction to calculate the averaged 2-dimensional (2D) map of the external validation dataset for all Transformer input feature maps of each class and the corresponding averaged Transformer output of each class. The difference between N and KC averaged Transformer's input maps was calculated and compared to the difference between averaged Transformer's N and K output to show how the Transformers modified this difference.^[26]^ To gain a deeper insight into the Transformer layers' performance in token space, we presented all of these averaged class-wise Transformer's input, output, and difference maps as heatmaps. Additionally, to assist visual detection of the difference between these heatmaps, we used the learned perceptual image patch similarity metric (LPIPSM) to generate the distance map between these heatmaps.^[27]^

### Statistical Analysis

All statistical analyses were performed using Scipy (Scientific Computing tools for Python, version 1.8.1)^[28]^ and scikit-learn libraries of Python (version 1.1.1).^[29]^ Descriptive statistical analysis was performed on baseline demographic characteristics, tomographic, corneal biomechanical parameters, and indices in all study groups. The probability distribution of each parameter was checked using the Kolmogorov–Smirnov one-sample test for goodness of fit.

The independent samples *t*-test was used to analyze parameters with normal distribution, while the Wilcoxon rank-sum test was used for nonparametric data to determine whether the data were significantly different between groups. For all analyses, *P* ≤ 0.05 was considered statistically signiﬁcant. The NBC was the machine learning algorithm chosen for solving this binary classification problem using the selected DCR parameters and indices. This model may be limited in its ability to represent complex datasets, but it effectively minimizes the risk of overfitting on relatively small datasets (such as our external validation dataset). Additionally, since NBC is quite simple, it typically converges faster than discriminative models like logistic regression.^[24]^

The Python programming language (version 3.9.13) was used to develop models. The Keras open-source software library (version 2.7.0) was used as an interface to the TensorFlow library (version 2.7.0). For the LPIPSM estimation, we used the PyTorch 1.0 + and torchvision libraries.

The sample size was calculated using NumPy (Numerical Python), a core Python scientific computing library. We implemented a NumPy t-test for two independent samples. Each group needed a sample size of at least 252 subjects (effect size = 0.25, alpha error = 0.05, power = 0.80).

A Python-based fast implementation of DeLong's algorithm was used for computing the statistical significance of comparing two AUCs.^[25]^ The pairwise correlation was calculated using the Spearman rank correlation coefficient. Deep-learning computations were performed on a single graphic processing unit (GPU).

##  RESULTS

A total of 821 eyes (392 right eyes and 429 left eyes) from 821 patients with KC and N participants were included. Table 1 shows the baseline characteristics of the study participants. Supplementary Figure 1 shows the BAD-D values of the study participants. There was no statistically significant difference in age or gender between the KC group and the N group in both datasets. Within the KC population in Datasets 1 and 2, there was a statistically significant difference between mean keratometry (Km), maximal keratometry (Kmax), CCT, and BAD-D. This ensures that the external validation dataset (Dataset 1) contains enough variability to assess model robustness in extracting characteristic features from unseen data.

**Figure 2:**
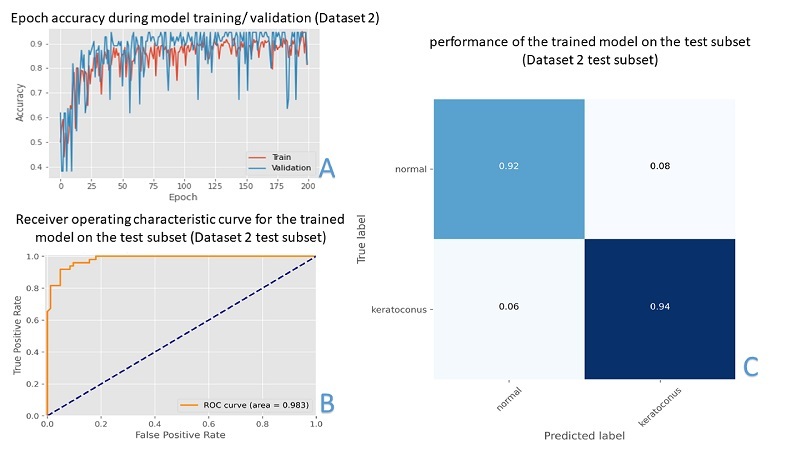
(A) Epoch accuracy during model training and internal validation (Dataset 2; Database 2 test subset). (B) Receiver operating characteristic (ROC) curve for binary (keratoconus versus normal) classification task by the trained model on the test subset (Dataset 2 test subset) with an area under the curve (AUC) of 0.983. (C) Confusion matrix showing the performance of the trained model on the test subset.

The Transformers-based classifier was trained for 200 iterations using feature maps extracted from the training and validation subsets (Dataset 2). Training progress is shown in Figure 2 along with the ROC curve and confusion matrix for model performance on the test subset (Dataset 2). The model accuracy on the test subset was 93% with an AUC of 0.983. The model sensitivity, specificity, precision, and F1 scores were 92%, 94%, 0.94, and 0.93 for the N group, respectively, and 94%, 92%, 0.92, and 0.93 for the KC group, respectively.

**Table 1:** Baseline and demographic data of the study population

**Parameter**	**Dataset 1**		***p***	**Dataset 2**		***p***	P*	P**
	**Normal**	**Keratoconus**		**Normal**	**Keratoconus**			
N	127	148	NA	285	261	NA		
Eye (right/left)	53/74	72/76	0.250	135/150	132/129	0.454	0.289	0.708
Gender (male/female)	66/61	64/84	0.148	119/166	124/137	0.176	0.054	0.405
Age (yrs)			0.054			0.092	0.184	0.073
Median	29	26		29	28			
IQR	8.0	9.0		9.0	9.5			
Range	19 to 51	18 to 52		20 to 52	19 to 53			
Km (D)			** < 0.001**			** < 0.001**	**0.041**	** < 0.001**
Median	43.82	46.65		43.2	48.38			
IQR	1.18	2.48		1.34	2.65			
Range	41.0 to 46.4	42.8 to 52.5		40.9 to 46.9	43.3 to 53.1			
Kmax (D)			** < 0.001**			** < 0.001**	**0.020**	**0.035**
Median	44.05	48.6		44.3	50.02			
IQR	0.96	2.55		1.28	3.14			
Range	41.8 to 47.6	45.2 to 56.6		41.5 to 47.7	44.5 to 58.8			
IOP (mmHg)			** < 0.001**			**0.002**	** < 0.001**	**0.025**
Median	17	14		14	15			
IQR	3.0	3.0		3.0	4.0			
PachySlope (µm)			** < 0.001**			** < 0.001**	** < 0.001**	** < 0.001**
Range	12 to 22	10 to 19		10 to 22	9 to 23			
CCT (µm)			** < 0.001**			** < 0.001**	** < 0.001**	** < 0.001**
Mean ± SD	523 ± 39.22	452 ± 42.13		538 ± 35.97	461 ± 47.66			
Range	463 to 602	378 to 531		457 to 613	390 to 500			
BAD-D			** < 0.001**			** < 0.001**	**0.004**	**0.021**
Median	0.65	8.8		0.85	9.18			
IQR	0.78	4.02		0.62	5.55			
Range	0.04 to 1.95	1.48 to 22.98		0.01 to 2.11	1.66 to 24.33			
A1 velocity [m/s]			** < 0.001**			** < 0.001**	0.061	**0.041**
Median	0.15	0.17		0.15	0.18			
IQR	0.2	0.2		0.3	0.02			
Range	0.12 to 0.18	0.12 to 0.20		0.11 to 0.19	0.15 to 0.21			
A1 DA [mm]			** < 0.001**			** < 0.001**	0.092	0.075
Median	0.09	0.11		0.1	0.12			
IQR	0.01	0.01		0.01	0.02			
Range	0.08 to 0.10	0.09 to 0.13		0.08 to 0.11	0.09 to 0.14			
DA ratio max (2 mm)			** < 0.001**			** < 0.001**	**0.041**	0.065
Median	4.52	5.75		4.15	6.1			
IQR	0.42	0.92		0.52	1.4			
Range	3.05 to 4.94	4.43 to 7.80		2.95 to 5.30	4.08 to 8.75			
Median	42.5	51.8		43.78	56.08			
IQR	8.08	20.88		10.18	26.12			
Range	26.44 to 62.32	44.90 to 106.78		25.40 to 78.70	40.60 to 118.35			
ARTh			** < 0.001**			** < 0.001**	** < 0.001**	** < 0.001**
Median	502.64	214.22		518.52	248.61			
IQR	144.22	175.87		168.22	238.22			
Range	220.30 to 708.45	102.27 to 490.16		192.88 to 803.28	84.31 to 544.92			
Integrated radius [mm ∧ –1]			** < 0.001**			** < 0.001**	** < 0.001**	** < 0.001**
Median	7.74	10.7		7.88	10.44			
IQR	0.94	2.22		1.41	3.15			
Range	6.83 to 8.54	8.54 to 14.21		5.93 to 9.54	8.02 to 18.56			
bIOP (mmHg)			** < 0.001**			** < 0.001**	** < 0.001**	**0.025**
Median	16.8	14.4		13.8	16			
IQR	3.2	3.7		3.5	3			
Range	12.9 to 22.1	10.8 to 19.2		11.3 to 21.7	9.4 to 23.2			
SP A1			** < 0.001**			** < 0.001**	**0.003**	** < 0.001**
Median	118.04	66.19		112.09	63.57			
IQR	32.15	27.67		28.28	22.34			
Range	71.36 to 149.68	20.35 to 142.36		69.25 to 152.11	18.67 to 147.33			
CBI			** < 0.001**			** < 0.001**	0.16	0.081
Median	0.01	0.95		0	0.97			
IQR	0.02	0.2		0.01	0.33			
Range	0.00 to 0.81	0.01 to 1.00		0.00 to 0.62	0.00 to 1.0			
TBI			** < 0.001**			** < 0.001**	0.454	0.052
Median	0	0.99		0.01	0.95			
IQR	0.03	0.15		0.05	0.11			
Range	0.09 to 0.75	0.96 to 1.0		0.06 to 0.80	0.85 to 1.0			
*Between Dataset 1 normal and Dataset 2 normal subgroup; **Between Dataset 1 keratoconus and Dataset 2 keratoconus subgroup. A1, first applanation; ARTh, Ambrósio relational thickness to the horizontal profile; BAD-D, Belin-Ambrósio enhanced ectasia total deviation index; bIOP, biomechanical intraocular pressure; CBI, Corvis Biomechanical Index; CCT, central corneal thickness; DA, deflection amplitude; IOP, intraocular pressure; IQR, interquartile range after removing outliers; Km, mean keratometry; Kmax, maximal apical keratometry; N, number of subjects; NA, not applicable; SP A1, stiffness parameter at first applanation; TBI, tomography and biomechanical index. Bold type signifies *P* ≤ 0.05.								

The mean inference time needed for a single video (including feature extraction) was 10.25 ± 0.33 seconds. This time can be reduced by preprocessing the first 95 frames to 6.91 ± 0.24. Furthermore, limiting the analysis to 10 frames (44 ${}^{\text{th}}$ to 53 ${}^{\text{rd}}$ frame) can reduce inference time to 1.07 ± 0.01 seconds on our single GPU.

We performed timing of the inference speed for the model variants to reveal the real-world speed of these architectures in our hardware.^[14]^ We experimented with building our feature extraction network with a backbone inherited from ResNet50V2^[30]^ at the end of stage 4 (before stage 5 MaxPooling) to generate feature maps with the shape of 139 × 1024. Using the exact Transformer dimensions, number of heads, and training recipe, we achieved a top accuracy of 87.12 with an inference time of 176 ms per extracted feature map. Additionally, we observed no improvement in accuracy as the number of tokens increased to 2048 when using the stage 5 output of ResNet50V2. Using the adopted DenseNet 121 model as a feature extractor, we varied the number of sampled video frames and observed a consistent improvement in accuracy as the number of frames increases (thereby increasing the number of tokens and temporal context). It was noted that the accuracy saturates after the 110 ${}^{\text{th}}$ frame, reflecting sufficient coverage to the main high-level concepts. However, the best performance (accuracy of 87.88%, inference time of 34 ms per extracted feature map) from the shortest frame sequence (10 frames) was obtained from the 44 ${}^{\text{th}}$ to 53 ${}^{\text{rd}}$ frames feature map, which appears reasonable in terms of accuracy/compute trade-off (Supplementary Videos 1–4).

Ablation of the Transformer encoder layers resulted in the worst performance (accuracy of 78.12%). Larger models with stacks of fully connected+ batch-normalization and more dropout layers worsened model performance and slowed inference. Also, using more Transformer encoder layers or increasing the number of heads using the same model dimension (d *${}_{model}$* of 1024) degraded model performance, and their implementation was extremely slow on our GPU. Using one Transformer encoder layer with one head or two layers with one head resulted in slightly lower accuracy (92.42% for both variants) with relatively low computational cost (inference time per feature map of 0.35 ms and 0.39 ms, consecutively). The specified training recipe was essential to unleash the potential of Transformers and prevent overfitting.

**Figure 3:**
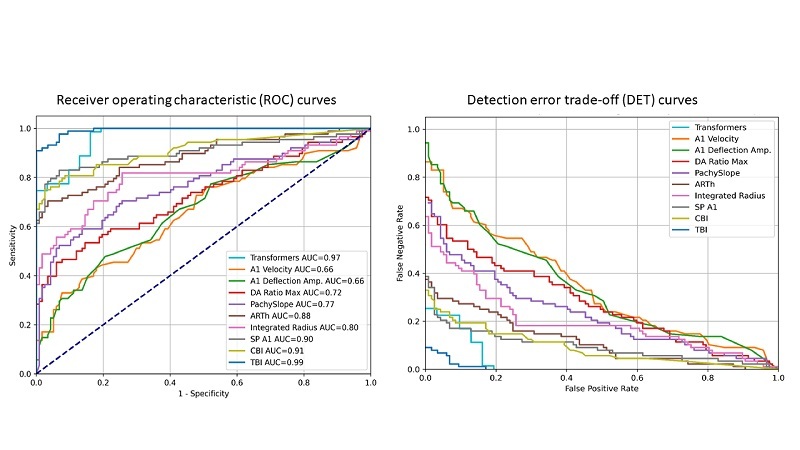
Receiver operating characteristic (ROC) curves and detection error trade-off curves for binary (keratoconus versus normal) classification task on the external validation subset (Dataset 1) by 9 Naïve Bayes classifiers, trained on A1 (first applanation) deflection amplitude in mm. A1 velocity, ARTh (Ambrósio relational thickness to the horizontal [8 mm] profile), Corvis biomechanical index (CBI), Deflection amplitude ratio at 2 mm (DA Ratio Max [2 mm]), Integrated Radius [mm ∧ -1], PachySlope (µm), stiffness parameter at first applanation (SP A1), and the tomographic and biomechanical index (TBI), compared to the performance of the trained Transformers-based classifier on feature maps extracted from the external validation subset (Dataset 1).

Tables 2 and 3 show the predictive accuracy and pairwise AUC comparisons of NBC(s) using the studied DCR parameters and summary indices of the external validation dataset (Dataset 1) compared to the performance of the Transformers-based classifier using the extracted feature maps from Corvis ST videos of the same dataset. Using DeLong's method, a pairwise comparison of NBC-based classifiers with the hybrid Transformers-based model showed that the TBI had the highest predictive accuracy, exceeding the Transformers-based classifier by a narrow, statistically significant margin. The performance of the Transformers-based model outperformed all other NBC classifiers with a statistically significant difference. The ROC and DET curves of all classifiers are depicted in Figure 3.

**Figure 4:**
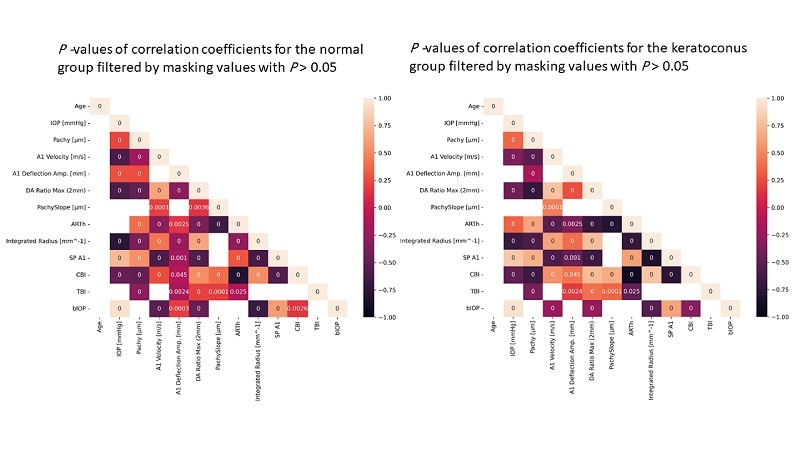
Heatmaps displaying Spearman rank correlation coefficients for normal and keratoconus groups after filtering for statistically significant correlations with *P*-value ≤ 0.05. A1 (first applanation); ARTh (Ambrósio relational thickness to the horizontal [8 mm] profile). bIOP, biomechanical intraocular pressure; CBI, Corvis biomechanical index; DA Ratio Max [2 mm] = deflection amplitude ratio at 2 mm; IOP, intraocular pressure; PachySlope (µm), Pachy, central corneal thickness; SPA1, stiffness parameter at first applanation; TBI, tomographic and biomechanical index.

Figure 4 shows the Spearman rank correlation analysis of the analyzed DCR parameters and indices with age, IOP, and CCT. Besides TBI, PachySlope, and ARTh were the only DCR parameters independent of the bIOP in both the N and KC groups. Also, bIOP had no significant correlation with age or central corneal thickness in both study groups.

**Figure 5:**
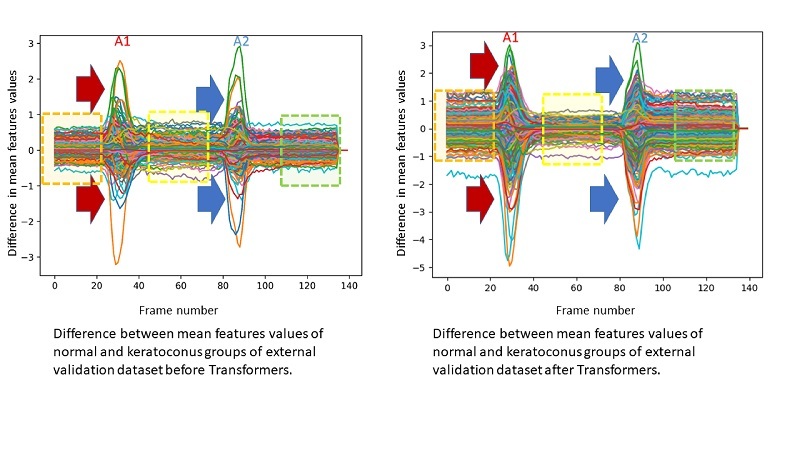
Difference between mean feature map values of all normal and keratoconus eyes in the external validation dataset before and after the Transformer layers. Orange, yellow, and green squares denote features before the first applanation (A1), between A1 and the second applanation (A2), and after A2, respectively. Arrows point to sparse features that were augmented by self-attention.

The difference between averaged N and KC Transformers' input feature maps and the corresponding Transformer's output is shown in Figure 5 and Supplementary Figure 2. The Transformers appear to successfully handle sparse representations within important feature domains by assigning more class-specific variability to learned values (before A1, at A1, at A2, and after A2), producing augmented feature maps for class prediction. Also, there are noticeably fewer differences in values assigned to class features in the interval between A1 and A2. Supplementary Figure 3 shows that the Transformers are judiciously allocating more values to features extracted from frames presenting A1 and A2 compared to other frames' features. This resulted in the enhancement of the difference between feature maps related to each class.

##  DISCUSSION

Using a Scheimpflug camera for noncontact ultra-high-speed corneal imaging has provided a new perspective for understanding corneal deformation processes through advanced image processing techniques. In softer, ectatic corneas, tangent modulus values have been reported to change.^[5]^ The static frames from the recorded slow-motion video are used to extract deformation response parameters.^[10]^

Inspired by the Transformers' success in modeling long-range contextual relationships in videos, we developed a hybrid model that extracts features from Corvis ST videos. These feature maps are converted into a compact set of tokens. These tokens are fed to Transformers to capture token interactions. The resulting augmented tokens can be directly used to discriminate between N and KC eyes. To the best of our knowledge, this is the first study that has used this technique to detect KC in Corvis ST corneal deformation videos without using corneal topography or tomography.

Increased accuracy in diagnosing KC has been demonstrated previously using machine learning algorithms based on corneal tomography parameters obtained by Pentacam or optical coherence tomography.^[31,32]^ However, a substantial body of literature suggests that the initiating event in KC is a focal reduction in biomechanical properties. The focal reduction in tangent modulus causes greater deformation in response to IOP, resulting in progressive corneal thinning and bulging.^[33]^ These findings have resulted in a rapid paradigm shift in strategies for early detection of KC to include assessments based on changes in pure biomechanical properties, which present before the appearance of secondary morphological manifestations.

Although ResNet is the most commonly used backbone network for video summarization tasks,^[34]^ our feature-extractor backbone variation experiment showed superior performance by DenseNet-121 feature maps compared to ResNet. Additionally, for each layer in DenseNet, the outputs (feature maps) of all previous layers are used as input to the next layer. This allows the network to work with very small output channel depths, which reduces the number of parameters.^[19]^

**Table 2:** External validation (Dataset 1) classification accuracy, precision, recall (sensitivity), f1 score, and receiver operating characteristic analysis with the AUC for the adopted trained hybrid model compared to Naïve Bayes classifiers' performance using the selected pachymetry and dynamic corneal response parameters and indices

**Parameter**	**Class**	**Recall**	**Specificity**	**Precision**	**F1 score**	**Accuracy**	**Mean AUC ± SE**
Hybrid model	Normal	0.84	0.93	0.92	0.87	0.88	0.970 ± 0.011
	Keratoconus	0.93	0.84	0.85	0.89		
A1 velocity [m/s]	Normal	0.90	0.33	0.57	0.70	0.61	0.667 ± 0.032
	Keratoconus	0.33	0.90	0.76	0.46		
A1 DA [mm]	Normal	0.99	0.11	0.52	0.68	0.61	0.662 ± 0.0.33
	Keratoconus	0.11	0.99	0.91	0.20		
DA ratio max (2 mm)	Normal	0.96	0.40	0.61	0.75	0.55	0.721 ± 0.031
	Keratoconus	0.40	0.96	0.90	0.55		
PachySlope (µm)	Normal	0.95	0.47	0.64	0.76	0.71	0.779 ± 0.028
	Keratoconus	0.47	0.95	0.90	0.62		
ARTh	Normal	0.95	0.70	0.64	0.84	0.82	0.883 ± 0.021
	Keratoconus	0.70	0.95	0.90	0.80		
Integrated radius [mm ∧ –1]	Normal	0.91	0.58	0.68	0.78	0.74	0.805 ± 0.027
	Keratoconus	0.58	0.91	0.86	0.69		
SP A1	Normal	0.96	0.75	0.79	0.87	0.86	0.905 ± 0.019
	Keratoconus	0.75	0.96	0.94	0.83		
CBI	Normal	0.97	0.78	0.81	0.88	0.87	0.910 ± 0.018
	Keratoconus	0.78	0.97	0.96	0.86		
TBI	Normal	0.95	0.93	0.93	0.94	0.94	0.994 ± 0.004
	Keratoconus	0.93	0.95	0.94	0.94		
AUC, area under the receiver operating characteristic curve; A1, first applanation; ARTh, Ambrósio relational thickness to the horizontal profile; CBI, Corvis Biomechanical Index; DA, deflection amplitude; SE, standard error; SP A1, stiffness parameter at first applanation; TBI, tomography and biomechanical index.							

**Table 3:** External validation (Dataset 1) ROC curves and AUC analysis for the Naïve Bayes classifiers' performance using the selected pachymetry and dynamic corneal response parameters and indices compared to the trained hybrid model

**Parameter**	**Pairwise comparison with the hybrid model using DeLong's method**			
	**Mean Δ AUC ± SE**	**95% CI**	**Z Statistic**	***P*** **-value**
A1 Velocity [m/s]	0.303 ± 0.012	0.261 to 0.344	8.753	** < 0.001**
A1 DA [mm]	0.308 ± 0.022	0.264 to 0.351	8.864	** < 0.001**
DA Ratio Max (2mm)	0.249 ± 0.020	0.0209 to 0.288	7.565	** < 0.001**
PachySlope (µm)	0.191 ± 0.017	0.0157 to 0.224	6.273	** < 0.001**
ARTh	0.087 ± 0.010	0.067 to 0.106	3.624	** < 0.001**
Integrated radius [mm ∧ –1]	0.165 ± 0.016	0.133 to 0.196	5.669	** < 0.001**
SP A1	0.065 ± 0.008	0.049 to 0.080	2.962	**0.003**
CBI	0.060 ± 0.007	0.046 to 0.073	2.755	**0.005**
TBI	0.024 ± 0.007	0.010 to 0.037	–2.006	**0.044**
** Δ ** AUC, difference between the area under the curve; A1, first applanation; ARTh, Ambrósio relational thickness to the horizontal profile; CBI, Corvis Biomechanical Index; CI, confidence interval; DA, deflection amplitude; ROC, receiver operating characteristic, SE, standard error; SP A1, stiffness parameter at first applanation; TBI, tomography and biomechanical index. Bold type signifies *P* ≤ 0.05.				

Following the training and testing of the adopted model on the Dataset 2 feature maps, its diagnostic capability to discriminate between N and KC eyes was assessed on the external validation subset (Dataset 1) feature maps to exclude overfitting. The model showed high sensitivity and specificity with an AUC of 0.97 in the external validation dataset, suggesting a promising generalization to unseen videos and confirming the diagnostic capability of our model. This performance was similar when we evaluated a subset of patients with mild KC. The hybrid model outperformed all other NBCs based on DCR parameters and indices except for the TBI, which remained the most robust index for distinguishing KC from N cases. This can be explained by the benefit of the integration of tomographic parameters in the calculation of this summary index.^[9]^ Also, TBI had no significant correlation with corneal thickness and IOP, the two confounding factors that have the greatest influence on *in vivo* corneal biomechanics. These findings are consistent with previous studies.^[35,36,37,38]^

Interestingly, the mean inference time per single video (including feature extraction) can be much reduced by preprocessing shorter frame sequences (down to 10 frames) without a dramatic reduction of accuracy. This relatively short inference time can allow real-time video classification during recording, but would require operating a high-performance GPU or a multi-threaded CPU.

Our limited ablation study confirmed the benefit of using multi-head attention as proposed in the original work by Vaswani et al.^[12]^ They hypothesized that the use of multi-head attention allows the model to jointly attend to information from different representation subspaces at different positions. However, using more than two heads did not improve performance in terms of accuracy. Of note, when using multi-head self-attention, the dimension of each head is reduced (head dimension = model dimension/number of heads) so that the total computational cost is similar to that of single-head attention with full dimensionality.

Ablation of the Transformer encoder layers resulted in the worst performance among all variations due to the inability to compute interactions between tokens. This finding validates the importance of capturing the relationship between different concepts in the feature maps and is consistent with earlier reports for visual Transformers when using graph convolutions or no token-space operations.^[11]^

In addition, our extensive experiments with various model variations and ablation showed that self-attention leads to systematic improvements in KC detection using features extracted from raw Corvis ST videos across a wide range of architectures and computational settings. This was consistent with findings of other studies assessing Transformers for image and video classification tasks.^[11,14,15]^

Plotting the difference between averaged N and KC Transformers input feature maps and the corresponding difference between averaged N and KC Transformer's output revealed the power of Transformers encoders to successfully handle sparse representations within important feature domains by assigning more class-specific variability to learned values (before A1, at A1, at A2, and after A2) producing augmented feature maps for class prediction. This can decrease the space and time complexity of the model. In addition, there are noticeably fewer differences in values assigned to class features in the interval between A1 and A2. This leveraging of sparsity by the Transformers resulted in noise smoothing, which prevents model overfitting by lowering model variance to insignificant (noisy) features with expected improvement in model performance in terms of accuracy/compute trade-off. We may also infer that the Transformers assigned lesser weights to features related to the corneal highest concavity for class prediction. This resulted in an overall enhancement of the difference between important tokens related to each class, while damping differences between unimportant (noisy) tokens.^[39]^ Leveraging such output map sparsity in inference incurs a negligible accuracy drop compared with the original network while accelerating inference when using fewer numbers of input frames. Recently, scaling Transformers using sparse layers has been used to develop efficient and faster models for unbatched decoding while scaling up the model size. Additionally, it can be implemented to increase prediction speed, in case of sampling all Corvis ST video frames.^[40]^

Herber et al^[38]^ used linear discriminant analysis and random forest algorithms to develop classification and staging models for KC using DCR and corneal thickness-related (pachymetry) parameters calculated by Corvis ST. Our approach cannot be directly compared to their work as we used the stand-alone Corvis ST videos without using either DCR parameters output by Corvis ST software or Pentacam's corneal topography or tomography data.

Recently, Tan et al^[41]^ used corneal contour data points extracted from each Corvis ST video frame to calculate parameters used to train a CNN for distinguishing N from KC. Compared to our studied population, they applied different inclusion criteria and used co-ethnic (Chinese) participants of a relatively smaller sample size that included some bilateral eyes. In contrast to their approach, we avoided employing the Otsu algorithm, as Otsu may generate errors in calculating numeric representations after simple corneal contour extraction. Furthermore, the traditional two-dimensional Otsu algorithm has several other drawbacks inherent to the use of the one-dimensional gray histogram of an image without considering the spatial information. In addition, this algorithm is affected by uneven brightness and noise, especially when considering the presence of multiple shadows and reflections from other anterior segment structures recorded in the Corvis ST video frames.^[42]^ Furthermore, our methodology can be exploited to extract corneal biomechanical information from all video frames rather than landmark frames.

Unlike Arnab et al,^[15]^ we did not use model regularization techniques. The use of strong regularization with an extensive list of combined augmentations can result in a massively inflated dataset with an enormous augmentation search space. Especially in domains with very limited data, this could result in further overfitting. Importantly, most of the available regularization techniques (e.g., random crop, flip, translations, color jitter, elastic distortions across scale, position, orientation flips, random cropping, erasing, or adding noise) can introduce various biases to the rigorous spatial-temporal parameters of medical datasets such as the Corvis ST videos, increasing the difficulty in identifying safe, label-preserving transformations.^[43]^

There was no analysis of our model's diagnostic potential in cases with subclinical KC, which is a limitation of this study. The sample size of patients with very asymmetric ectasia defined as the presence of obvious KC in one eye based on clinical or topographic characteristics and a topographically normal fellow eye (that possibly represents subclinical KC) was fewer than the needed population to allow comparison of these topographically normal eyes with N and KC groups in terms of statistical power. Thus, we included only the ectatic eye of these patients in the KC group. Although most of the parameters of the N and KC groups were statistically different [Table 1], there was a significant overlap between the parameters of the eyes in the N and KC groups. Notably, well over 20% of the yes in the validation dataset were either suspect or early-stage KC (based on BAD D parameter), and our model consistently detected these eyes with suspect and early KC, similar to more established cases.

In future work, we will consider testing the capabilities of our model to separate N eyes from eyes with subclinical KC on a sufficient sample size that permits reliable statistical power. In addition, we suggest using a pooling-based tokenizer to spatially sub-sample feature maps to reduce their spatial dimension, permitting a lower computational cost. Additionally, given the synchronized release of all the 139 Corvis ST video frames in response to the similar spatiotemporal profile of the air puff, the extracted feature maps should represent synchronized embedded feature semantics. This allows the implementation of cluster-based tokenization (e.g., k-means clustering) of extracted features to group these features into a pseudo-semantic space, which can reduce the temporal dimension of the features from 139 to a lower number of tokens, thereby reducing computation.^[11,15]^

##  CONCLUSION

In conclusion, our study introduces a hybrid Transformers-based CNN model for KC diagnosis from stand-alone Corvis ST videos. The model shows high sensitivity and specificity on the test subset and external validation data from another continent. Our results suggest the possible use of this algorithm in everyday clinical practice to augment the current KC diagnostic armamentarium without requiring corneal topography imaging. Additional independent data is needed to validate the model's performance within other population characteristics.

##  Financial Support and Sponsorship

This research was conducted by the Cornea Research Network (CRN) initiative, an international effort to develop artificial intelligence models to address corneal conditions, and received no specific grant from any funding agency in the public, commercial, or not-for-profit sectors.

##  Conflicts of Interest

None.

## References

[1] Rabinowitz YS. Keratoconus. *Surv Ophthalmol* 1998;42:297–319. 10.1016/s0039-6257(97)00119-7 9493273

[2] Labiris G, Giarmoukakis A, Sideroudi H, Gkika M, Fanariotis M, Kozobolis V. Impact of keratoconus, cross-linking and cross-linking combined with photorefractive keratectomy on self-reported quality of life. *Cornea* 2012;31:734–739. 10.1097/ICO.0b013e31823cbe85 22236781

[3] Fernández Pérez J, Valero Marcos A, Martínez Pe na FJ. Early diagnosis of keratoconus: What difference is it making? *Br J Ophthalmol* 2014;98:1465–1466. 10.1136/bjophthalmol-2014-305120 PMC4215270 24759873

[4] Pi nero DP. Technologies for anatomical and geometric characterization of the corneal structure and anterior segment: A review. *Semin Ophthalmol* 2015;30:161–170. 10.3109/08820538.2013.835844 24175646

[5] Scarcelli G, Besner S, Pineda R, Yun SH. Biomechanical characterization of keratoconus corneas ex vivo with Brillouin microscopy. *Invest Ophthalmol Vis Sci* 2014;55:4490–4495. 10.1167/iovs.14-14450 PMC4109405 24938517

[6] Roberts CJ, Dupps WJ Jr. Biomechanics of corneal ectasia and biomechanical treatments. *J Cataract Refract Surg* 2014;40:991–998. 10.1016/j.jcrs.2014.04.013 PMC4850839 24774009

[7] Roberts CJ, Mahmoud AM, Bons JP, Hossain A, Elsheikh A, Vinciguerra R, et al. Introduction of two novel stiffness parameters and interpretation of air puff-induced biomechanical deformation parameters with a dynamic Scheimpflug analyzer. *J Refract Surg* 2017;33:266–273. 10.3928/1081597X-20161221-03 28407167

[8] Vinciguerra R, Ambrósio R Jr, Elsheikh A, Roberts CJ, Lopes B, Morenghi E, et al. Detection of keratoconus with a new biomechanical index. *J Refract Surg* 2016;32:803–810. 10.3928/1081597X-20160629-01 27930790

[9] Ambrósio R Jr, Lopes BT, Faria-Correia F, Salom ao MQ, Bühren J, Roberts CJ, et al. Integration of Scheimpflug-based corneal tomography and biomechanical assessments for enhancing ectasia detection. *J Refract Surg* 2017;33:434–443. 10.3928/1081597X-20170426-02 28681902

[10] Ali NQ, Patel DV, McGhee CN. Biomechanical responses of healthy and keratoconic corneas measured using a noncontact Scheimpflug-based tonometer. *Invest Ophthalmol Vis Sci* 2014;55:3651–3659. 10.1167/iovs.13-13715 24833745

[11] Bichen W, Chenfeng X, Dai X, Wan A, Zhang P, Tomizuka M, et al. Visual Transformers: Token-based image representation and processing for computer vision. ArXiv; 2020. https://doi.org/10.48550/arXiv.2006.03677

[12] Vaswani A, Shazeer N, Parmar N, Uszkoreit J, Jones L, Gomez AN, et al. Attention is all you need. ArXiv; 2017. https://doi.org/10.48550./arXiv.1706.03762

[13] Bello I, Zoph B, Vaswani A, Shlens J, Le QV. Attention augmented convolutional networks. ArXiv; 2019. https://doi.org/10.48550./arXiv.1904.09925

[14] Dosovitskiy A, Beyer L, Kolesnikov A, Weissenborn D, Zhai X, Unterthiner T, et al. An image is worth 16x16 words: Transformers for image recognition at scale. ArXiv; 2020. https://doi.org/10.48550./arXiv.2010.11929

[15] Arnab A, Dehghani M, Heigold G, Sun C, Lučić M, Schmid C. ViViT: A video vision Transformer. ArXiv; 2021. https://doi.org/10.48550./arXiv.2103.15691

[16] Krumeich JH, Daniel J, Knülle A. Live-epikeratophakia for keratoconus. *J Cataract Refract Surg* 1998;24:456–463. 10.1016/s0886-3350(98)80284-8 9584238

[17] Joda AA, Shervin MM, Kook D, Elsheikh A. Development and validation of a correction equation for Corvis tonometry. *Comput Methods Biomech Biomed Engin* 2016;19:943–953. 10.1080/10255842.2015.1077515 27049961

[18] Xu X, Lin J, Tao Y, Wang X. An improved DenseNet method based on transfer learning for fundus medical images. *7th International Conference on Digital Home (ICDH)* 2018:137–140.

[19] Zhang C, Benz P, Argaw DM, Lee S, Kim J, Rameau F, et al. ResNet or DenseNet? Introducing dense shortcuts to ResNet. ArXiv; 2020. https://doi.org/10.48550./arXiv.2010.12496

[20] Deng J, Dong W, Socher R, Li LJ, Li K, Fei-Fei L. Imagenet: A large-scale hierarchical image database. *IEEE Conference on Computer Vision and Pattern Recognition* 2009:248–255.

[21] Goodfellow I, Bengio Y, Courville A. Deep learning. MIT Press; 2016.

[22] Ruder S. An overview of gradient descent optimization algorithms. ArXiv; 2016. https://doi.org/10.48550./arXiv.1609.04747.

[23] Powers DM. Evaluation: From precision, recall, and F-measure to ROC, informedness, markedness & correlation. *J Mach Learn Technol* 2011;2:37–63.

[24] Webb GI. Naïve Bayes. In: Sammut C, Webb GI, editors. Encyclopedia of machine learning. Springer; 2011.

[25] Sun X, Xu W. Fast implementation of DeLong's algorithm for comparing the areas under correlated receiver operating characteristic curves. *IEEE Signal Process Lett* 2014;21:1389–1393.

[26] Jolliffe IT, Cadima J. Principal component analysis: A review and recent developments. *Philos Trans A Math Phys Eng Sci* 2016;374:20150202. 10.1098/rsta.2015.0202 PMC4792409 26953178

[27] Zhang R, Isola P, Efros AA, Shechtman E, Wang O. The unreasonable effectiveness of deep features as a perceptual metric. ArXiv; 2018. https://doi.org/10.1109/CVPR.2018.00068

[28] Oliphant TE. Python for scientific computing. *Comput Sci Eng* 2007;9:10–20.

[29] Pedregosa F, Varoquaux G, Gramfort A, Gramfort A, Michel V, Thirion B, et al. Scikit-learn: Machine learning in Python. *J Mach Learn Res* 2011;12:2825–2830.

[30] Rahimzadeh M, Attar A. A modified deep convolutional neural network for detecting COVID-19 and pneumonia from chest X-ray images based on the concatenation of Xception and ResNet50V2. *Inform Med Unlocked* 2020;19:100360. 10.1016/j.imu.2020.100360 PMC7255267 32501424

[31] Smadja D, Touboul D, Cohen A, Doveh E, Santhiago MR, Mello GR, et al. Detection of subclinical keratoconus using an automated decision tree classification. *Am J Ophthalmol* 2013;156:237–246.e1. 10.1016/j.ajo.2013.03.034 23746611

[32] Shi C, Wang M, Zhu T, Zhang Y, Ye Y, Jiang J, et al. Machine learning helps improve diagnostic ability of subclinical keratoconus using Scheimpflug and OCT imaging modalities. *Eye Vis* 2020;7:48. 10.1186/s40662-020-00213-3 PMC7507244 32974414

[33] Scarcelli G, Besner S, Pineda R, Yun SH. Biomechanical characterization of keratoconus corneas ex vivo with Brillouin microscopy. *Invest Ophthalmol Vis Sci* 2014;55:4490–4495. 10.1167/iovs.14-14450 PMC4109405 24938517

[34] Elharrouss O, Akbari Y, Almaadeed N. Backbones-review: Feature extraction networks for deep learning and deep reinforcement learning approaches. ArXiv; 2022. https://doi.org/https://doi.org/10.48550./arXiv.2206.08016

[35] Ferreira-Mendes J, Lopes BT, Faria-Correia F, Salom ao MQ, Rodrigues-Barros S, Ambrósio R Jr. Enhanced ectasia detection using corneal tomography and biomechanics. *Am J Ophthalmol* 2019;197:7–16. 10.1016/j.ajo.2018.08.054 30201341

[36] Kataria P, Padmanabhan P, Gopalakrishnan A, Padmanaban V, Mahadik S, Ambrósio R Jr. Accuracy of Scheimpflug-derived corneal biomechanical and tomographic indices for detecting subclinical and mild keratectasia in a South Asian population. *J Cataract Refract Surg* 2019;45:328–336. 10.1016/j.jcrs.2018.10.030 30527442

[37] Wu Y, Guo LL, Tian L, Xu ZQ, Li Q, Hu J, et al. Comparative analysis of the morphological and biomechanical properties of normal cornea and keratoconus at different stages. *Int Ophthalmol* 2021;41:3699–3711. 10.1007/s10792-021-01929-4 PMC8536578 34232432

[38] Herber R, Ramm L, Spoerl E, Raiskup F, Pillunat L, Terai N. Assessment of corneal biomechanical parameters in healthy and keratoconic eyes using dynamic bidirectional applanation device and dynamic Scheimpflug analyzer. *J Cataract Refract Surg* 2019;45:778–788. 10.1016/j.jcrs.2018.12.015 30902432

[39] Li Z, You C, Bhojanapalli S, Li D, Rawat AS, Reddi SJ, et al. Large models are parsimonious learners: Activation sparsity in trained Transformers. ArXiv; 2022. https://doi.org/https://doi.org/10.48550./arXiv.2210.06313

[40] Jaszczur S, Chowdhery A, Mohiuddin A, Kaiser Ł, Gajewski W, Michalewski H, et al. Sparse is enough in scaling Transformers. ArXiv; 2021. https://doi.org/https://doi.org/10.48550./arXiv.2111.12763.\

[41] Tan Z, Chen X, Li K, Liu Y, Cao H, Li J, et al. Artificial intelligence-based diagnostic model for detecting keratoconus using videos of corneal force deformation. *Transl Vis Sci Technol* 2022;11:32. 10.1167/tvst.11.9.32 PMC9527334 36178782

[42] Wang W, Duan L, Wang Y. Fast image segmentation using two-dimensional Otsu based on estimation of distribution algorithm. *J Electr Comput Eng* 2017;2017:1735176.

[43] Shorten C, Khoshgoftaar TM. A survey on image data augmentation for deep learning. *J Big Data* 2019;6:1–48. 10.1186/s40537-021-00492-0 PMC8287113 34306963

